# Intravesical BCG treatment causes a long-lasting reduction of recurrence and progression in patients with high-risk non-muscle-invasive bladder cancer

**DOI:** 10.1007/s00345-018-2375-7

**Published:** 2018-06-15

**Authors:** Tomas Thiel, Charlotta Ryk, Lotta Renström-Koskela, Gunnar Steineck, Martin C. Schumacher, N. Peter Wiklund, Petra J. de Verdier

**Affiliations:** 1 Department of Urology, Karolinska University Hospital, Stockholm, Sweden; 2 Urology Laboratory, Department of Molecular Medicine and Surgery, Karolinska Institutet, Stockholm, Sweden; 3 Department of Clintec, Karolinska Institutet, Stockholm, Sweden; 4 Department of Oncology-Pathology, Karolinska Institutet, Stockholm, Sweden; 5 Division of Clinical Chemistry, Department of Laboratory Medicine, Karolinska Institutet, Karolinska University Hospital, 141 86 Stockholm, Sweden; 6 Norra Kungsvägen 11b, 18131 Lidingö, Sweden

**Keywords:** Non-muscle-invasive bladder cancer, BCG, Survival, Progression, Recurrence

## Abstract

**Purpose:**

To analyse if BCG treatment leads to long-term reduction of recurrence, progression, and cancer-specific mortality (CSM) in patients with high-risk NMIBC.

**Materials and methods:**

140 patients with high-risk NMIBC were drawn from a population-based cohort of 538 patients with newly diagnosed bladder cancer in the Stockholm County between 1995 and 1996. Data were collected prospectively, and a final follow-up for recurrence, progression, and CSM was performed after 15 years. Patients that received BCG were compared with patients who did not receive BCG. Survival analysis was done with Kaplan–Meier estimates and Mantel–Cox log-rank test. Multivariable Cox proportional regression with stepwise selection was performed to verify the statistical significance of clinicopathological factors of prognostic importance. Results were displayed in Hazard ratios and a *p *< 0.05 was considered to be statistically significant.

**Results:**

With a median follow-up of 100 months (2–182), 76 patients recurred; 50 progressed to muscle invasion; and 92 died of whom 38 died from bladder cancer. After 15-year follow-up, there was a statistically significant reduction in rate for recurrence (HR 0.40, *p* < 0.0001) and progression (HR 0.52, *p = *0.038), but not for CSM, in patients that received BCG compared to those who did not.

**Conclusions:**

In this group, BCG in high-risk NMIBC patients reduced the long-term risk of recurrence and progression. The effect on CSM is yet to be clarified.

## Introduction

Intravesical Bacillus Calmette et Guerin (BCG) is an established treatment for non-muscle-invasive bladder cancer (NMIBC) and was introduced in 1976 by Morales [1]. The European association of Urology (EAU) recommends that all high-risk NMIBC patients should receive intravesical instillations of BCG [2]. According to a working group of EAU guidelines for NMIBC, high-risk NMIBC consists of all stage T1-, TaG3-, primary, and concomitant cancer in situ of the bladder (CIS) and recurrent and large TaG1G2 tumours [3]. EORTC developed risk tables to calculate a risk of recurrence and progression in an individual patient with NMIBC (1 and 5 years). Based on these probabilities, EAU working group suggested to stratify tumours in to three groups (low, intermediate, and high) to reflect the risk of both recurrence and progression using recurrence/progression score [4].

For high-risk NMIBC tumours, the risk of progression and cancer-specific mortality (CSM) is increased as compared to TaG1–G2 tumours [5]. Important factors associated with a high risk of recurrence are tumour size and number; important factors for progression and disease-specific survival are tumour grade and stage [2]. The 5-year progression rate for patients with T1 tumours ranges from 6 to 40% [4]. In patients with CIS, the corresponding rate is slightly higher (54%) [6].

BCG is given as intravesical instillations [1] and the recommended treatment regimen includes an induction course of 6 weekly instillations followed by additional instillations for up to 36 months [3, 7]. While it is established that intravesical BCG reduces the number of recurrent NMIBC [8], there is an uncertainty whether BCG treatment prevents or delays progression to muscle invasion or metastases, or decreases CSM. While several studies report a decrease of progression and CSM after BCG treatment [9–11], a study by Malmström et al. from 2009 [8] suggests no such effect. Cochrane reports from 2003 and 2011 failed to see any effect of BCG on progression or cancer-specific mortality. Nevertheless, these reports indicate that BCG is superior to other intravesical agents, e.g., mitomycin and epirubicin, in reducing tumour recurrence [12, 13]. It has been shown that BCG reduces recurrence in CIS patients [14, 15]. In BCG maintenance treatment for CIS, the complete response rate is as high as 84%, although the 5-year disease free survival is lower (70%) due to extravesical recurrence and progression [14].

The objective of this study was to analyse if BCG treatment reduces recurrence, progression, and CSM in patients with high-risk NMIBC. A 15-year clinical follow-up has been performed in a population-based study material consisting of the majority of patients with newly diagnosed high-risk NMIBC in the Stockholm county in 1995–1996 (*n* = 140).

## Materials and methods

### Patient cohort and clinical assessment

Over a period of 2 years, 1st of January 1995 to 31st of December 1996, all patients newly diagnosed with urothelial carcinoma (UC) in the Stockholm County were asked to participate in a prospective cohort study. In total of 538 patients, 76% (538/705) of all cases in the county during that period, agreed to participate. The entire cohort was described in a 5-year clinical follow-up published in 2003 [5] and in an additional article in 2010 [16]. From this patient material, all cases with high-risk NMIBC were included in the present study, a total of 140 patients. The same pathologist performed all histopathological assessments and the standard pathological report included data on stage, grade, presence of concomitant CIS, and detrusor muscle in the specimen. The tumour, node, and metastasis (TNM) classification from 1978 was used for histopathological classification [17]. For tumour grading, the WHO 1999 malignancy grading system was applied [18]. High-risk NMIBC was defined as all non-muscle-invasive T1 and all G3 tumours, e.g., T1G1–G3, TaG3, and primary and secondary CIS, according to EAU standards [19] and previous results from our group [5].

The initial transurethral resection (TUR-BT) for staging and local control was performed in all patients. Treatment options for high-risk NMIBC were at that time TUR-BT only, or with adjuvant intravesical chemo- or immunotherapy. A second resection was not performed on a regular basis and only recommended when tumour size > 3 cm. Data on residual tumours in this procedure were not registered systematically. Any additional treatment was offered to the discretion of the treating physician according to the local traditions and the patient’s performance status and request, however. Immediate cystectomy was not standard and performed only in occasional cases (*n* = 3). Multidisciplinary conference was recommended but not performed for all newly diagnosed bladder cancers.

BCG immunotherapy (Connaught 2 × 10^8^ to 3 × 10^9^ bacteria/instillation) was given according to the manufacturer’s recommendations as an induction course of 6 weekly instillations. Maintenance treatment, either monthly instillations or 3 weekly instillations every 3 months, (for at least 1 year), was recommended but not given according to a single standard. Data on the duration of BCG-treatment were not registered during the 15-year clinical follow-up and due to different archive systems in the participating hospitals it has not been possible to obtain this information retrospectively. Cystoscopy and cytology were included in the routine follow-up (every 3 months for the first year and every 6 months for the second year and thereafter annually for 3 years). A CT scan was performed only when there were symptoms of progression.

### Follow-up

Patient records of all original participants were scrutinized in 2011. Follow-up time was defined as the time elapsed from date of diagnosis to death or last clinical evaluation. Parameters registered initially were as follows: date of diagnosis, sex, age, stage, grade, tumour size, multifocality, presence of concomitant CIS, and presence of detrusor muscle in the resected material. Parameters collected continuously and at the end of the study were: number and date of recurrence, date of progression to muscle-invasive tumour, development of lymph-node and distant metastasis, type of therapy, and date of and cause of death. EORTC score for risk of recurrence and progression was calculated, according to the methods defined by EORTC [2] based on the initial data, and categorized as of intermediate or high risk. In cases where cause of death was registered as bladder cancer, without any previous progression, date of progression was considered to be the same as date of death. All patients included in the 5-year clinical follow-up were also included in the follow-up after 15 years.

Diagnosis of any new tumour in the bladder after the initial staging and grading by TUR-BT was considered as recurrence. Growth of the initial tumour into the muscular layer of the bladder or beyond, local, and distant metastasis or death from bladder cancer was considered as progression. Cancer-specific death was defined as death from bladder cancer according to the Swedish cause of death register.

### Statistical analysis

The present study was performed on a fixed prospective cohort. Patients who received BCG were compared with patients who did not, in terms of time to recurrence, progression, and CSM. Univariable analysis for each of these terms was done for Kaplan–Meier survival estimates and curves were compared using the Mantel–Cox log-rank test. Multivariable Cox proportional regression with stepwise selection was used to verify the prognostic significance of clinicopathological factors of importance (age, sex, stage, grade, tumour size, multiple tumours, and concomitant CIS). Results were expressed as hazard ratios and a *p* < 0.05 was considered statistically significant. In many studies, primary CIS is considered to be a separate entity. In the present study, all primary CIS patients were given BCG, and separate analysis both with and without CIS were, therefore, carried out. Finally, as a sensitivity analysis, all patients with CIS (primary and concomitant) were excluded to examine the impact of BCG on papillary tumours alone, a condition in which BCG adjuvant treatment is secondary only to TUR-BT. Statistical analysis was performed with IBM^®^ SPSS^®^ Statistics, version 21.0 software.

## Results

### Cohort data

In total, 140 patients were enrolled in the study. One patient was excluded due to inconsistent information on survival. Median age was 71.5 years and the ratio male:female was 2.2:1. Among the 139 patients’ tumours, 78% were staged as T1, 14% as Ta and 8% as primary CIS. Predominant grade was G3 (60%). Size > 3 cm was common (64%), while multifocality was less frequent (43%). According to EORTC scores for recurrence, most patients were at intermediate risk, while EORTC scores for progression indicated high risk for > 90% of the patients. Median follow-up was 100 months and none of the patients were lost after the first 5-year follow-up. BCG-treated patients were younger (68.5 vs. 72 years), were staged less as T1 (68 vs. 93%), had more primary CIS (13 vs. 0%) and concomitant CIS (34 vs. 5%), and had more G3 tumours (71 vs. 46%) than non-BCG-treated patients. Detrusor muscle was obtained in 83% of the patients with T1 tumours, and there was no significant difference between BCG-treated and non-BCG-treated patients (86 vs 83%) (see Table 1).

**Table 1:** Baseline characteristics and follow-up of the 139 high-risk NMIBC patients

Variable	BCG, *n* (%)	Non-BCG, *n* (%)	Total, *n* (%)	*p*
No. (%)	82 (59)	57 (41)	139 (100)	0.002
Age, year (median)	68.5	72	71.5	0.02
Sex				
Male	58 (71)	38 (67)	96 (69)	0.61
Female	24 (29)	19 (33)	43 (31)	
Tumour stage				
Ta	15 (18)	4 (7)	19 (14)	0.06
T1	56 (68)	53 (93)	109 (78)	0.005
CIS	11 (13)	0	11 (8)	0.004
Concomitant CIS^a^				
Y	24 (34)	3 (5)	27 (21)	0.0001
N	47 (66)	54 (95)	101 (79)	
Grade				
G1	3 (4)	3 (5)	6 (4)	0.69
G2	21 (25)	28 (49)	49 (36)	0.002
G3	58 (71)	26 (46)	84 (60)	0.003
No. tumours^a^				
1	43 (61)	30 (53)	73 (57)	0.37
≥ 2	28 (39)	27 (47)	55 (43)	
Size^a^				
< 3 cm	26 (37)	20 (35)	46 (36)	0.85
≥ 3 cm	45 (63)	37 (65)	82 (64)	
Presence of detrusor (for stage T1 tumours)				
Y	48 (86)	44 (83)	91 (83)	0.70
N	8 (14)	9 (17)	18 (17)	
EORTC score progression^a^				
Intermediate	6 (9)	6 (11)	12 (9)	0.69
High	65 (91)	51 (89)	116 (91)	
EORTC score recurrence^a^				
Intermediate	63 (89)	53 (93)	116 (91)	0.41
High	8 (11)	4 (7)	12 (9)	
Follow-up				
Range, months	2–182	4–182	2–182	
Median follow-up, months	121	108	100	
Mean follow-up, months	108	81	97	0.0001

*BCG* Bacillus Calmette–Guérin, *CIS* carcinoma in situ, *EORTC* The European Organization for research on treatment of cancer

^a^Primary CIS not included

The initial treatment in all cases was TUR-BT. Only 14 patients received a non-BCG adjuvant intravesical instillation. There were three cases of immediate cystectomy, while deferred cystectomy due to recurrence or progression was performed in 25 patients (18%). Cystectomy was more common in non-BCG patients, while other treatment modalities were not (see Table 2).

**Table 2:** Treatment other than BCG

	BCG, *n* (%)	Non-BCG, *n* (%)	Total, *n* (%)	*p*
Curative intention				
Instillation (mitomycin)	8 (10)	6 (11)	14 (10)	0.88
Cystectomy	10 (12)	15 (27)	25 (18)	0.03
Immediate cystectomy	0	3 (5)	3 (2)	0.04
Radiotherapy	3 (4)	3 (5)	6 (4)	0.65
Chemotherapy	0	3 (5)	3 (2)	0.04
Adj/neoadj chemotherapy	2 (2)	2 (4)	4 (3)	0.71
Palliation				
Salvage cystectomy	1 (1)	1 (2)	2 (1)	0.79
Radiotherapy	3 (4)	8 (14)	11 (8)	0.02
Chemotherapy	0	2 (4)	2 (1)	0.09

Within 5 years of clinical follow-up, 29 patients (21%) had died of urothelial carcinoma. Almost all patients who died of bladder cancer, 37 out of 38 patients, had died within 10 years from diagnosis. After 15 years of clinical follow-up, 92 patients (66%) had died, of which 38 patients (27%) died of urothelial carcinoma. Stage progression was seen in 50 patients (36%) and recurrence in 76 patients (55%) (see Table 3).

**Table 3:** Clinical outcome *n* (%)

	BCG	Non-BCG	Total	*p*
Recurrence				
Yes	35 (42.7)	41 (71.9)	76 (54.7)	0.001
No	47 (57.3)	16 (28.1)	63 (45.3)	
Progression to T2NM				
Yes	21 (25.6)	29 (50.9)	50 (36.0)	0.002
No	61 (74.4)	28 (48.1)	89 (64.0)	
CSM				
Yes	16 (19.5)	22 (39.0)	38 (27.3)	0.01
No	66 (80.5)	35 (61.0)	101 (72.7)	
Overall death				
Yes	50 (61.0)	42 (73.7)	92 (66.2)	0.42
No	32 (39.0)	15 (26.3)	47 (33.8)	

*BCG* Bacillus Calmette–Guérin, *T2NM* stage 2–4, lymph-node metastasis or distant metastasis or CSM, *CSM* cancer-specific mortality

### Outcome from BCG treatment

**Fig. 1:**
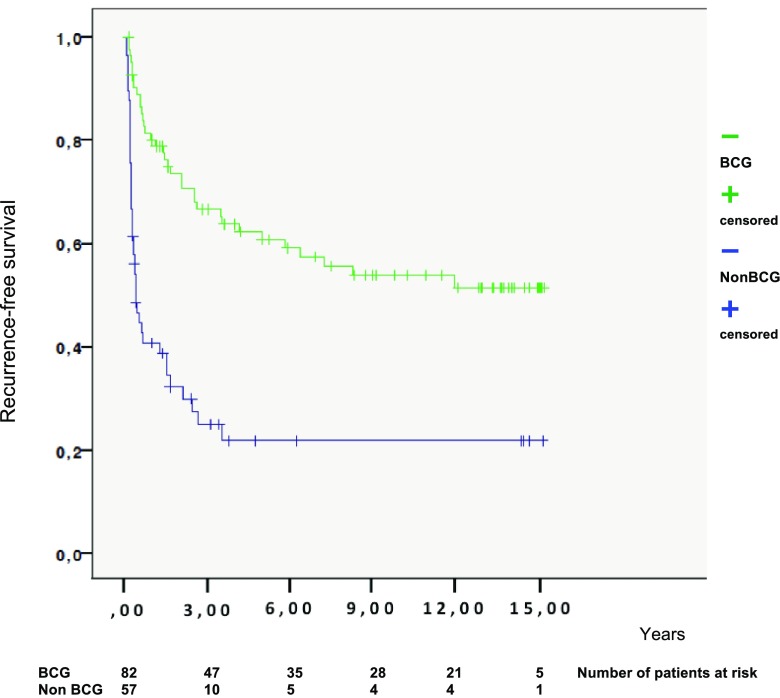
Recurrence-free survival according to treatment (BCG or not). *BCG* Bacillus Calmette–Guérin

Patients that received BCG treatment had less recurrence than the group that did not receive BCG [hazard ratio (HR) 0.40, *p* < 0.0001]. In addition, after exclusion of patients with primary CIS tumours (*n* = 11), there was a statistically significant difference between the two groups (HR 0.40, *p* < 0.0001) (see Table 4 and Fig. 1).

**Table 4:** Univariable and multivariable analyses of time to recurrence

	Univariable		Multivariable	
	HR (95% CI)	*p* value	HR (95% CI)	*p* value
All patients				
Non-BCG	1.0		1.0	
BCG	0.30 (0.19–0.48)	< 0.0001	0.40 (0.24–0.65)	< 0.0001
Ex. CIS				
Non-BCG	1.0		1.0	
BCG	0.34 (0.21–0.54)	< 0.0001	0.40 (0.24–0.65)	< 0.0001

Multivariable analysis after stepwise selection: age and concomitant CIS

*BCG* Bacillus Calmette–Guérin, *CI* confidence interval, *HR* hazard ratio, *CIS* cancer in situ

**Fig. 2:**
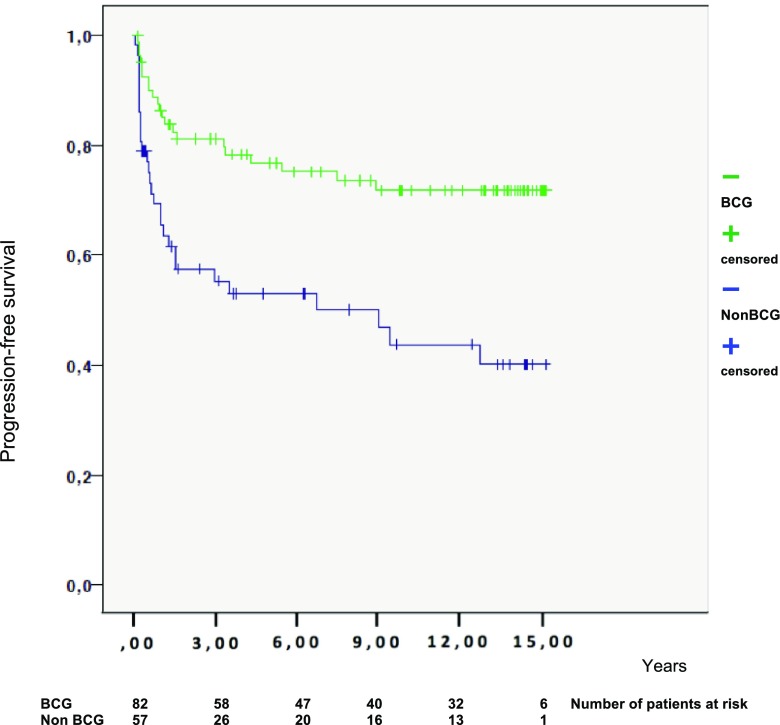
Progression-free survival according to treatment (BCG or not). *BCG* Bacillus Calmette–Guérin

For progression, the results showed a statistically significant difference between the BCG-treated and the non-BCG-treated group, both before (HR 0.52, *p* = 0.038) and after exclusion of patients with primary and concomitant CIS (HR 0.41, *p* = 0.018). However, after exclusion of primary CIS patients, significance was lost after adjustments for age and stage (HR 0.58 *p**** = ***0.075) (see Table 5 and Fig. 2).

**Table 5:** Univariable and multivariable analyses of time to progression

	Univariable		Multivariable	
	HR (95% CI)	*p* value	HR (95% CI)	*p* value
All patients				
Non-BCG	1.0		1.0	
BCG	0.38 (0.22–0.67)	0.001	0.52 (0.28–0.97)	0.038
Ex. CIS				
Non-BCG	1.0		1.0	
BCG	0.41 (0.23–0.72)	0.002	0.58 (0.32–1.06)	NS (0.075)
Ex CIS and concomitant CIS				
Non-BCG	1.0		1.0	
BCG	0.30 (0.15–0.61)	0.001	0.41 (0.20–0.86)	0.018

Multivariable analysis after stepwise selection: age, concomitant CIS, and stage

*BCG* Bacillus Calmette–Guérin, *CI* confidence interval, *HR* hazard ratio, *CIS* cancer in situ, *NS* non-significant

**Fig. 3:**
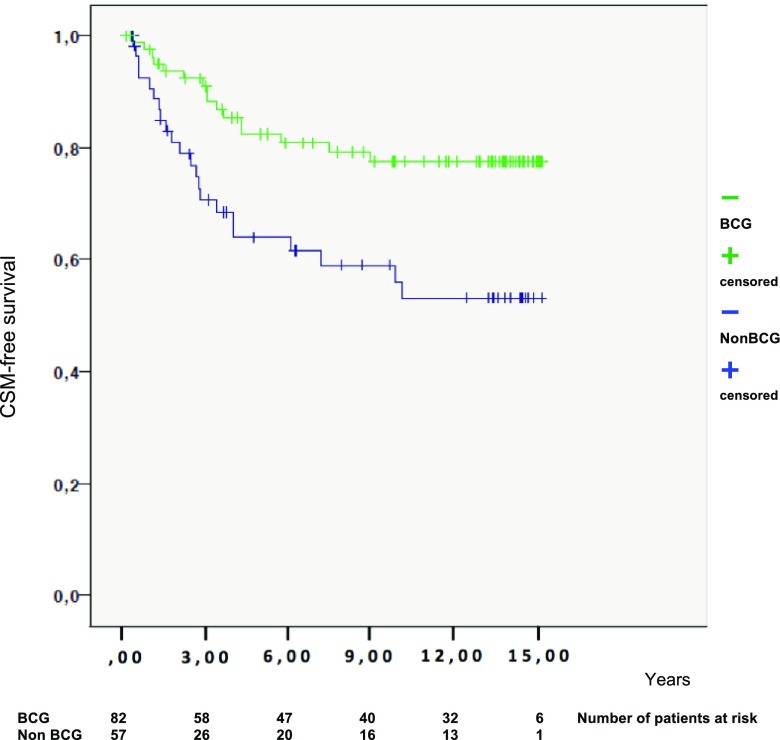
CSM-free survival according to treatment (BCG or not). *CSM* cancer-specific mortality, *BCG* Bacillus Calmette–Guérin

Death from urothelial carcinoma occurred at a lower rate in the BCG-treated group as compared to the non-BCG-treated group [20 vs. 39%, HR 0.40, *p* = 0.006 (see Table 3)]. However, after adjustments for age, stage, tumour size, and number of tumours, no statistically significant difference could be measured (see Table 6 and Fig. 3.).

**Table 6:** Univariable and multivariable analyses of time to CSM

	Univariable		Multivariable	
	HR (95% CI)	*p* value	HR (95% CI)	*p* value
All patients				
Non-BCG	1.0		1.0	
BCG	0.40 (0.21–0.77)	0.006	0.58 (0.29–1.16)	NS
Ex. CIS				
Non-BCG	1.0		1.0	
BCG	0.45 (0.24–0.86)	0.016	0.78 (0.39–1.55)	NS
Ex CIS and concomitant CIS				
Non-BCG	1.0		1.0	
BCG	0.46 (0.22–0.94)	0.034	0.75 (0.35–1.62)	NS

Multivariable analysis after stepwise selection: age and stage

*BCG* Bacillus Calmette–Guérin, *CI* confidence interval, *CSM* cancer-specific mortality, *HR* hazard ratio, *CIS* cancer in situ, *NS* non-significant

## Discussion

In this study, we investigated the long-lasting effect of BCG in patients with high-risk NMIBC, on treatment outcome in terms of recurrence, progression, and CSM. There was a strong statistically significant difference in recurrence between patients that received BCG treatment and the patients that did not, in favour of BCG treatment. In a study by Patard et al. [20], the recurrence rate was 50% for TUR-B + BCG vs 90% for TUR-B only. This result is similar to ours despite a longer follow-up in our study, probably reflecting observations from other studies [21] that most recurrence events in NMIBC patients occur within 5 years from primary diagnosis. In a retrospective study by Shahin et al. including only patients with T1G3 tumours, there was only a small difference in recurrence between the BCG-treated and the non-BCG-treated group (70 vs. 75%) [22]. The patients in our study were more heterogenic in stage and grade and the difference in the estimated effect as compared to Shahin et al. most likely reflects T1 tumours being more aggressive than high-grade Ta tumours [2]. Thus, several studies have shown that BCG reduces recurrence in high-risk NMIBC, and the results in the present study confirm this notion.

Limitations when it comes to measure efficacy of BCG on tumour progression include heterogeneity of patient populations, a short follow-up period of most studies along with a nonuniform definition of disease progression [9, 21]. In the present study we had a well-defined group of patients (high-risk NMIBC), a long follow-up (15 years) and progression defined as an increase to stage T2 or higher or presence of lymph-node and distant metastases. With these criteria fulfilled, there was a statistically significant reduction of progression in the group of BCG-treated patients, which suggests that intravesical BCG has a long-lasting effect on progression. However, it has previously been suggested that BCG is less effective against T1G3 tumours with concomitant CIS [22], and in line with that, we did not find a significant association with BCG treatment and decrease of progression in this group. For patients with primary CIS, a comparison with the non-BCG-treated group was not possible in our study, since all primary CIS patients received BCG. However, in the present study, only 1 out of 11 (9%) patients with primary CIS progressed. This result contrasts with the previous observations that untreated CIS has a progression rate of over 50% [14], and suggests a positive effect of BCG-treatment. Another observation in the present study was the association between BCG treatment and a decrease in progression in patients with high-risk papillary tumours without concomitant CIS. Previously published studies by Patard et al. [20] and Sylvester et al. [11] have shown similar effects.

CSM in our study affected 27% of the cohort. CSM in the BCG-treated group of patients was almost half that seen in the non-BCG-treated group. 80% of the deaths occurred within 5 years from diagnosis and only one death occurred later than 10 years from diagnosis. Overall mortality was 66% during the 15-year follow-up. Despite a well-defined patient cohort in a prospective study with a long follow-up and a high death rate as indicated above, there was no statistically significant difference between the two treatment groups following multivariable analysis when primary CIS was excluded. This finding corroborate with the fact that no randomized study has shown a reduction in cancer-specific mortality in patients with high-risk NMIBC after the initial treatment with BCG. One explanation might be the influence of competing mortality as indicated by the high rate of overall mortality in the present study and the small sample size of only 140 patients.

In our study, 36% of the patients suffered from progression. In the group of patients who were given intravesical BCG, progression rate was lower (26%), but still the rates are comparatively high. In a multicenter study of NMIBC by Cambier et al. [23] and in retrospective studies of high-risk NMIBC by Baniel et al. [24] and Hurle et al. [25], the progression rates were 20, 8, and 18% respectively. In these studies, however, the median follow-up was shorter (60, 56 and 85 months) as compared to the present study (121 months) and only BCG-treated patients with T1G3 tumours were included.

Detrusor muscle presence in first TUR-BT is considered to be a surrogate marker of resection quality that predicts risk of the early recurrence [26]. In our study, detrusor muscle was present in 83% of the T1 tumours, indicating a high resection quality in Stockholm county in 1995 and 1996. However, there was no significant difference between BCG-treated patients and non-BCG-treated patients in the presence of detrusor muscle that would explain any difference of outcome between the two groups.

Our study was a prospective cohort study with a non-randomized inclusion of almost all high-risk NMIBC patients within a specified residence area during 24 months. Although the patient group that received BCG differed slightly, in terms of higher degrees of high-grade and Ta and CIS tumours, from the patient group that did not receive BCG, the EORTC scores for risk of recurrence and progression were similar between the two groups at base line. To compensate for the differences between the two groups, we performed multivariable analysis, but there might still be confounders (e.g., smoking) that we have not been able to compensate for. In addition, several previous studies [8, 21] have shown that maintenance treatment increases the effect of BCG on recurrence and progression. Unfortunately, from the clinical data, for this study, we were not able to obtain information about length and frequency of BCG treatment. BCG treatment was recommended for these patients, and when there was a decision not to use BCG, this was based on local traditions and the request and performance status of every patient. Particular reasons for not giving BCG treatment were not registered, however.

## Conclusion

The present study supports the previous findings that intravesical treatment with BCG in patients with high-grade NMIBC decreases recurrence, regardless of maintenance treatment and a number of risk factors, e.g., grade and stage. We also show that BCG reduces progression in papillary tumours without concomitant CIS. The effect on recurrence and progression was long-lasting and the results of our study support the established recommendation of using BCG as the first-line adjuvant treatment in patients with high-risk NMIBC, especially in patients with primary CIS or papillary tumours without concomitant CIS. The question of whether BCG reduces CSM or not remains to be solved.
